# *Trypanosoma cruzi* Infection through the Oral Route Promotes a Severe Infection in Mice: New Disease Form from an Old Infection?

**DOI:** 10.1371/journal.pntd.0003849

**Published:** 2015-06-19

**Authors:** Juliana Barreto-de-Albuquerque, Danielle Silva-dos-Santos, Ana Rosa Pérez, Luiz Ricardo Berbert, Eliane de Santana-van-Vliet, Désio Aurélio Farias-de-Oliveira, Otacilio C. Moreira, Eduardo Roggero, Carla Eponina de Carvalho-Pinto, José Jurberg, Vinícius Cotta-de-Almeida, Oscar Bottasso, Wilson Savino, Juliana de Meis

**Affiliations:** 1 Laboratory on Thymus Research, Oswaldo Cruz Institute, Oswaldo Cruz Foundation, Rio de Janeiro, Brazil; 2 Immunology Institute, Faculty of Medical Science, National University of Rosario, Rosario, Argentina; 3 Laboratory on Molecular Biology and Endemic Diseases, Oswaldo Cruz Institute, Oswaldo Cruz Foundation, Rio de Janeiro, Brazil; 4 Laboratory on Experimental Pathology, Immunobiology Department, Federal Fluminense University, Niteroi, Brazil; 5 National and International Laboratory on Triatomine Taxonomy, Oswaldo Cruz Institute, Oswaldo Cruz Foundation, Rio de Janeiro, Brazil; Universidade Federal de Minas Gerais, BRAZIL

## Abstract

Oral transmission of Chagas disease has been documented in Latin American countries. Nevertheless, significant studies on the pathophysiology of this form of infection are largely lacking. The few studies investigating oral route infection disregard that inoculation in the oral cavity (Oral infection, **OI**) or by gavage (Gastrointestinal infection, **GI**) represent different infection routes, yet both show clear-cut parasitemia and heart parasitism during the acute infection. Herein, BALB/c mice were subjected to acute **OI** or **GI** infection using 5x10^4^ culture-derived *Trypanosoma cruzi* trypomastigotes. **OI** mice displayed higher parasitemia and mortality rates than their **GI** counterparts. Heart histopathology showed larger areas of infiltration in the **GI** mice, whereas liver lesions were more severe in the **OI** animals, accompanied by higher Alanine Transaminase and Aspartate Transaminase serum contents. A differential cytokine pattern was also observed because **OI** mice presented higher pro-inflammatory cytokine (IFN-γ, TNF) serum levels than **GI** animals. Real-time PCR confirmed a higher TNF, IFN-γ, as well as IL-10 expression in the cardiac tissue from the **OI** group compared with **GI**. Conversely, TGF-β and IL-17 serum levels were greater in the **GI** animals. Immunolabeling revealed macrophages as the main tissue source of TNF in infected mice. The high mortality rate observed in the **OI** mice paralleled the TNF serum rise, with its inhibition by an anti-TNF treatment. Moreover, differences in susceptibility between **GI** *versus* **OI** mice were more clearly related to the host response than to the effect of gastric pH on parasites, since infection in magnesium hydroxide-treated mice showed similar results. Overall, the present study provides conclusive evidence that the initial site of parasite entrance critically affects host immune response and disease outcome. In light of the occurrence of oral Chagas disease outbreaks, our results raise important implications in terms of the current view of the natural disease course and host-parasite relationship.

## Author Summary

Chagas disease caused by the protozoan *Trypanosoma cruzi* is endemic in Latin America and a neglected tropical disease, which affects 6–7 million people worldwide. Currently, oral transmission is the most frequent pathway of infection in Brazil but also occurs in other endemic countries. This important infection route is underestimated and understudied. Here, we demonstrate that the site of parasite entrance, in the oral cavity (**OI**), as observed in natural infection, or directly to the gastrointestinal tract (**GI**), differentially affects the host-immune response and mortality. **OI** promotes a severe acute disease, elevated parasitemia and TNF mediated mortality. **OI** showed intense hepatitis and mild heart damage. Interestingly, **GI** mice presented mild disease, along with less circulating TNF and higher TGF-β and IL-17 serum contents. **GI** animals showed mild liver damage and intense heart inflammation. Our study is a pioneer work that analyzes the features of two distinct routes of oral infection. In addition, it provides new clues for Chagas pathology and stimulates background for the elucidation of disease features in orally exposed populations.

## Introduction

Chagas disease (American trypanosomiasis), caused by the protozoan *Trypanosoma cruzi*, affects 6–7 million people worldwide, with an annual incidence of 28 thousand cases in the Americas [WHO, 2015]. Chagas disease is endemic in 21 countries in Latin America and was previously confined to this region. However, it has spread to other continents due to the migration of infected people [1]. Transmission to humans occurs through excreta deposition after biting of contaminated insect vectors belonging to the *Reduviidae* family, blood transfusion, organ transplantation, laboratory accident as well as congenitally and orally [2,3].

The first case of *T*. *cruzi* oral transmission in Brazil was reported in 1965 in Teotonia, Rio Grande do Sul [4]. Since then, outbreaks of orally transmitted Chagas disease have occurred in several Brazilian states, such as Amazonas, Amapá, Bahia, Ceará, Pará, Paraíba, Rio Grande do Sul and Santa Catarina. Although underestimated, oral transmission of Chagas disease was responsible for more than 739 cases in the Pará State in legal Amazônia, Brazil (1986–2012); 369 cases in Venezuela (2007–2009); 45 cases in Colombia (2008–2010); 14 cases in Bolivia (2010), and orally transmitted Chagas disease was reported in Argentina and Ecuador [5,6,7,8,9,10,11]. All of these outbreaks were associated with food/beverages consumption like: wild infected animal meat, vegetables, sugar cane extract, açaí pulp, goiaba juice, bacaba, babaçu and vino de palma [5,12,13,14]. Interestingly, oral transmission of Chagas disease is currently the most important transmission pathway in the Brazilian Amazon after the pan-American Health Organization declared the interruption of vectorial transmission in this area [6]. In past years, the proportion of orally infected patients has increased. From 1968 to 2000, 50% of acute cases in the Amazon region were attributed to oral transmission,[9] and between 2000–2010, the rate reached 70% [7]. Furthermore, wild strains of the parasite in oral transmission lead to cardiac involvement in patients in the Amazon region [14,15,16,17]. Mortality rates in these orally infected patients are higher (8–35%) when compared with the classical vectorial transmission through triatomine excreta deposition after biting (<5–10%) [18]. Despite this, there are controversies about mortality rates, because oral transmission gained more attention after outbreaks over the years. Previous studies suggested that metacyclic trypomastigotes are more infective by oral than cutaneous challenge, emphasizing the higher severity of oral infection [19].

The common immunological knowledge of experimental *T*. *cruzi* infection comes from studies with intraperitoneal/ subcutaneous infected mice [20,21]. Although relevant, there are few reports regarding *T*. *cruzi* oral transmission in the literature. Some authors have demonstrated parasite-mucosa interaction and few aspects of immune response and disease outcome after intragastric, pharyngeal or oral cavity parasite challenge. These models of oral *T*. *cruzi* infections result in parasitemia and heart parasitism, which indicates systemic infection [22,23,24,25,26]. In addition, *T*. *cruzi* glycoproteins (e.g., gp82) seem to bind gastric mucin, promoting invasion and replication in epithelial cells from the gastric mucosa [27]. This initial invasion is related to the establishment of a progressive gastritis and allowing further systemic dissemination of the parasite. Nonetheless, the short replication period at this mucosal site induces specific immunity, as protection was observed after a secondary mucosal challenge, involving the production of IgA and IgG antibodies [23]. Interestingly, humoral and cellular responses are also protective after parasite inoculation in the conjunctival mucosa, a natural portal of entry for *T*. *cruzi* that leads to nasal infection with subsequent systemic spreading [28]. In orally infected mice, inflammatory infiltrates are observed in several tissues, such as the pancreas, spleen, liver, bone marrow, heart, duodenum, adrenal glands, brain and skeletal muscle [23]. Moreover, it was suggested that intraepithelial and lamina propria lymphocytes are involved in IFN-γ, but not IL-4 production, in orally infected hosts [23]. Interestingly, this infection route does not affect CD8^+^ T cell response [26]. Following disease outbreaks caused by food contamination with *T*. *cruzi*, a clear increase in the severity of clinical manifestations was observed in these infected patients compared with other types of transmission routes [9,18]. These observations raise important questions concerning the particular features of *T*. *cruzi* entry via the mucosa, including the possible modulation of local immune mechanisms and the impact on regional and systemic immunity [20,21]. Herein, we demonstrate that the site of parasite entrance, through the oral cavity (as observed in natural infection- **OI**) or directly into the stomach (**GI**), differentially affects host immune response and mortality. In this study, we demonstrate that a highly severe acute disease follows in mice subjected to **OI**, compared with **GI**. They presented elevated parasitemia, high TNF serum levels, hepatitis and mild carditis, as well as a high mortality rate, which were partly reverted by anti-TNF therapy. This pioneer study approaches two distinct routes of oral infection that not only provides new clues for understanding Chagas pathology but also stimulates background for the elucidation of disease features in orally exposed populations.

## Results

### OI-infected mice present higher parasitemia and mortality compared with GI infection

**Fig 1:**
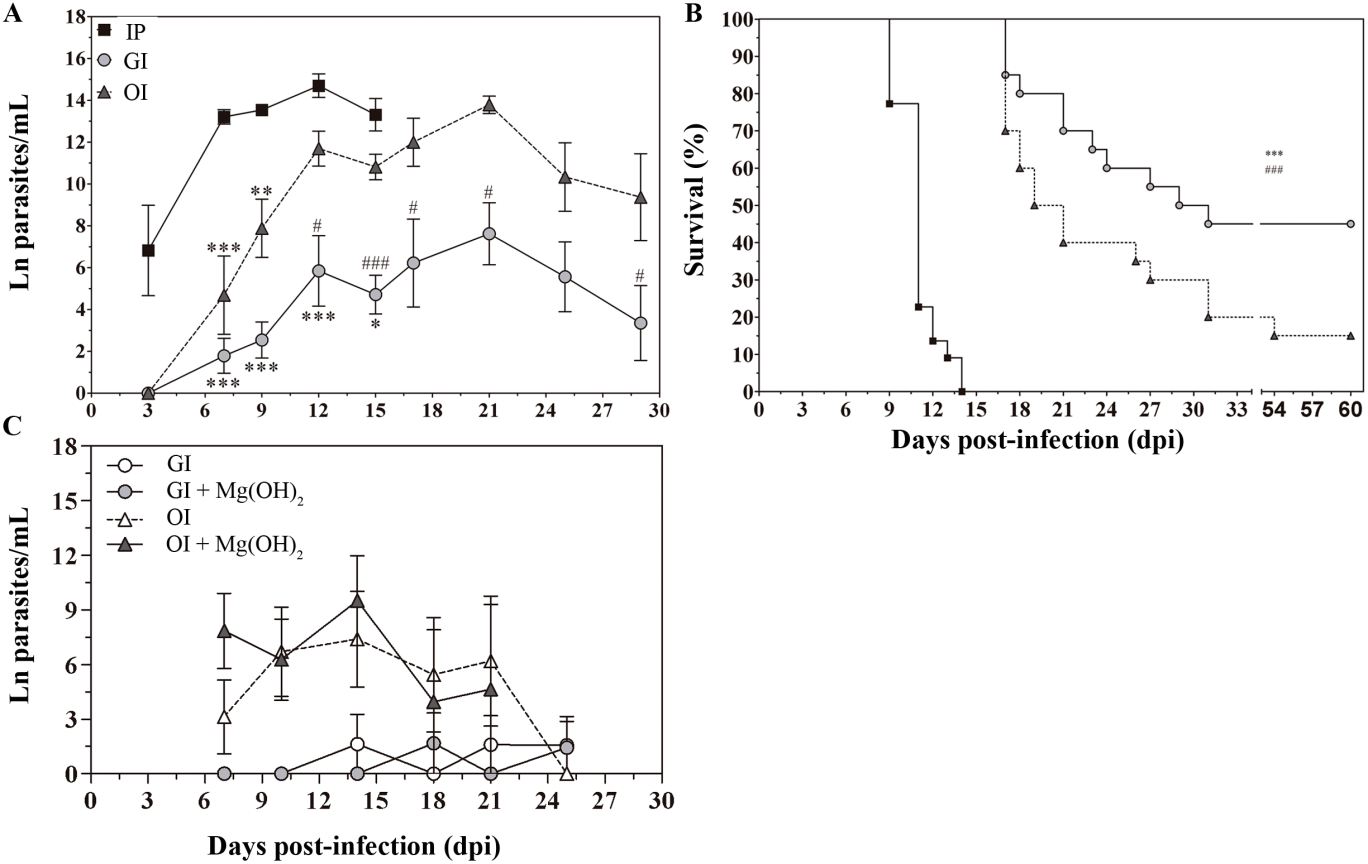
Severity of acute *T*. *cruzi* infection is higher in orally infected mice. A/B) Male BALB/c mice were infected with 5x10^4^ tissue culture-derived trypomastigotes forms of *T*. *cruzi* (Tulahuén strain) through gavage (GI) or oral cavity (OI). C) GI and OI *T*. *cruzi* inoculation was performed with antacid (Magnesium Hydroxide suspension, 19.4 mg/Kg) or medium. A/C) Parasitemia (mean and SEM) was assessed during the acute phase and expressed as ln parasites per milliliter for statistical analysis. Parasites were counted by light microscopy, and parasitemia calculated by the Brener method. Parasitemia comparisons were performed at different days post-infection (dpi), Kruskal-Wallis, Dunn’s post-test (until 15 dpi) and one-tailed Mann-Whitney (after 15 dpi) tests were used. A) n: IP, 3 dpi = 3, 7 dpi = 17, 9 dpi = 10, 12 dpi = 5, 15 dpi = 3; GI, 3dpi = 7; 7 dpi = 22; 9 dpi = 29; 12 dpi = 17; 15 dpi = 45; 17 dpi = 10; 21 dpi = 24; 25 dpi = 16; 29 dpi = 11 and OI, 3 dpi = 4; 7 dpi = 9; 9 dpi = 14; 12 dpi = 22; 15 dpi = 40; 17 dpi = 12; 21 dpi = 14; 25 dpi = 8; 29 dpi = 6. Lower numbers represent early stages, when parasitemia was still undetectable and final stages, when mortality rates were too high. The total number was obtained from different experiments. * represent differences in comparison to IP and #, differences between GI and OI. C) GI = 7 and OI = 7 from Mg(OH)_2_ treated mice and controls. B) Mortality was followed and survival was analyzed by Log-rank (Mantel-Cox) (*) and Gehan-Breslow-Wilcoxon (#) tests. n = 20 mice (equivalent to 100%). Statistical analysis was performed using GraphPad Prism 5. * p = 0.05; ** p = 0.01; *** p = 0.001.

BALB/c mice were infected with the highly virulent *T*. *cruzi* Tulahuén strain (DTU- TcVI). In order to assess whether the route of infection interferes in the course of infection, infectivity, mortality and parasitemia were analyzed in intragastrically **(GI)**, oral cavity/orally **(OI)** or intraperitoneally **(IP)** infected mice (Fig 1A and 1B and S1 Fig). **IP** infection, with 5x10^4^ trypomastigotes promoted elevated infectivity, parasitemia and mortality (Figs 1 and S1). Regarding the mucosal pathway of infection, **OI** mice were more susceptible to *T*. *cruzi* infection than **GI** mice, with higher parasitemia, mortality (Fig 1A and 1B) and infectivity (97.5% and 49.3%, respectively) (S1 Fig).

Differences in the infectivity rate may be associated with the low stomach pH, affecting parasite burden or its molecules. In our model of infection, mice were kept without water and food for 4 hours, and at that moment, the gastric pH was 3 and the oral cavity pH was 5. Treatment with the antacid Magnesium Hydroxide (Mg(OH)_2_ Phillips—19.4 mg/Kg) immediately neutralized the stomach pH to 7 and maintained the gastric pH at 5 for 30 minutes. In our study, differences in parasitemia observed between **GI** and **OI** could not be attributed to the acidic gastric pH in the mucosa because the Mg(OH)_2_ suspension addition at the time of inoculation (pH = 7) in both experimental groups did not interfere with blood-parasite burden (Fig 1C). Antacid treatment five minutes before infection showed similar results.

Taken together, our data clearly demonstrate that *T*. *cruzi* trypomastigote exposure in the oral cavity leads to a highly severe acute disease in mice. Moreover, although **GI** and **OI** are considered similar mucosal infection routes, their pattern of host response is not the same.

### GI-infected mice present more extensive cardiac tissue compromise, whereas OI infection leads to significant hepatic lesions

**Fig 2:**
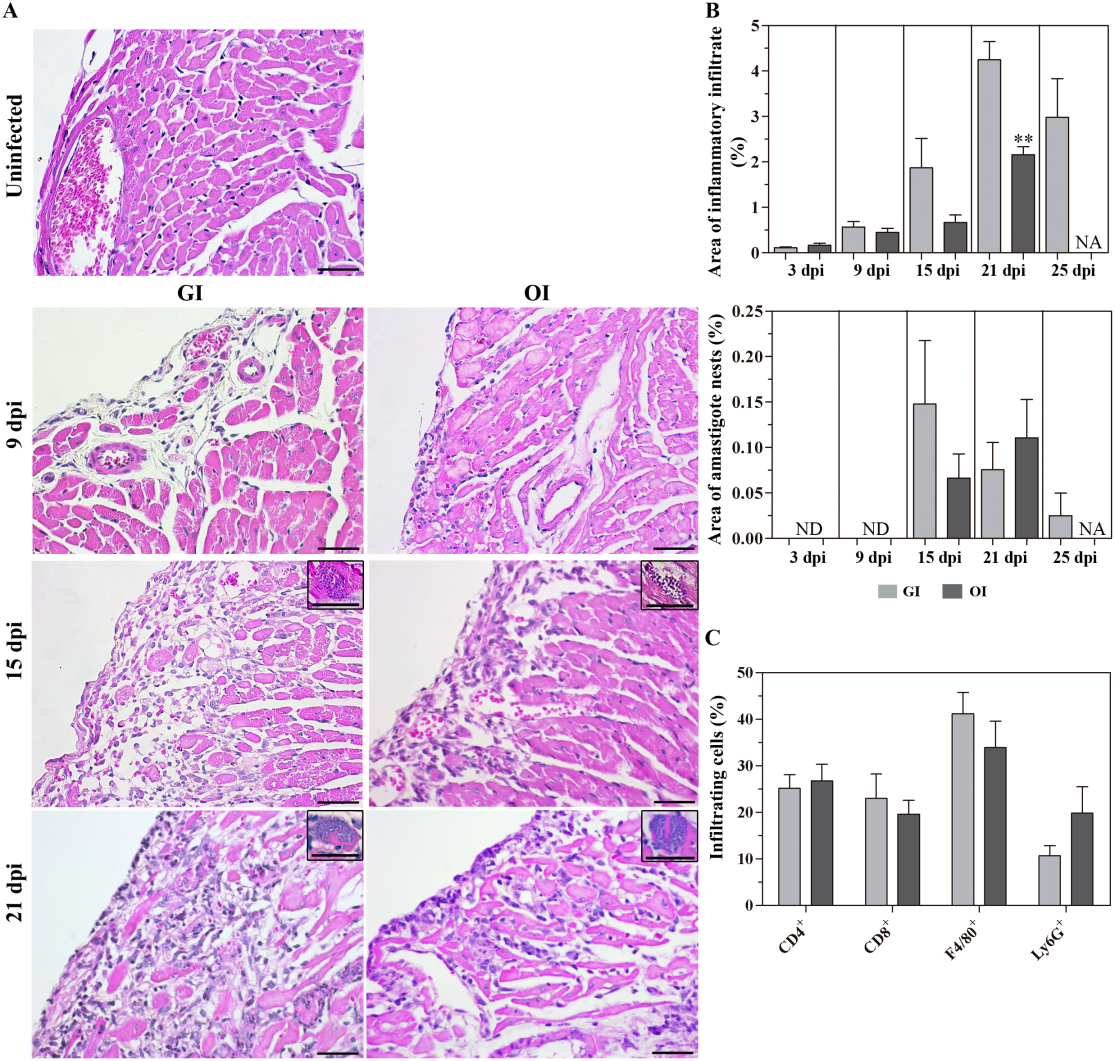
Hearts of GI infected mice are more inflamed than the OI infected mice. Male BALB/c mice were infected with 5x10^4^ tissue culture-derived trypomastigotes forms of *T*. *cruzi* (Tulahuén strain) through gavage (GI) or oral cavity (OI). Hearts were harvested at different days post-infection (dpi), fixed and embedded in paraffin. A) Histological longitudinal sections were stained by Hematoxylin-Eosin. For the quantification of inflammatory infiltrate and amastigotes nests in heart tissue, a relative area of infiltrate/or amastigote nests from 50 fields (400X) was analyzed. Pictures represents cells-rich infiltrated areas. B) Values are the mean ± SEM. n = 4–5 mice/dpi/group. *GI *versus* OI. C) Immunofluorescence analyses demonstrating the percentage of each subset present within the tissue after 16/17 dpi, CD4^+^, CD8^+^, F4/80^+^ and Ly6G^+^ cells. Numbers represent mean±SEM. n = 3 mice/group (two different sections from each mouse). Statistical analysis was performed using GraphPad Prism 5. Comparison between GI and OI groups was performed by using one-tailed Mann-Whitney test. * p = 0.05; **p = 0.01; ***p = 0.001. dpi, days post-infection. Ui, uninfected. N.A., not analyzed. Bars represent 20 μm. Inserts show amastigote nests.

The myocardium is one of the most affected tissues during *T*. *cruzi* infection in patients [18]. As we observed that different inoculation routes could distinctly affect acute phase severity, a histopathological analysis of heart sections was performed in 3, 9, 15, 21 and 25 dpi (days post-infection). At initial stages of infection (3–9 dpi), scarce infiltration is observed in the pericardium of both **GI** and **OI** groups (S3 Table). Nevertheless, inflammatory infiltration was significantly higher in the **GI**-infected mice than in **OI** after 15 dpi, affecting both the pericardium and the myocardium (Fig 2A and 2B and S3 Fig). Mild collagen deposition was observed in both groups when compared with uninfected mice (S3 Fig).

In conformity with previous studies in these experimental models, **IP**-infected mice showed extensive inflammatory infiltration in the heart throughout the course of the acute phase [29]. As observed in Fig 2, both groups showed a similar profile of infiltrating cells (CD4 and CD8 cells, F4/80^+^ macrophages and Ly6G^+^ neutrophils).

**Fig 3:**
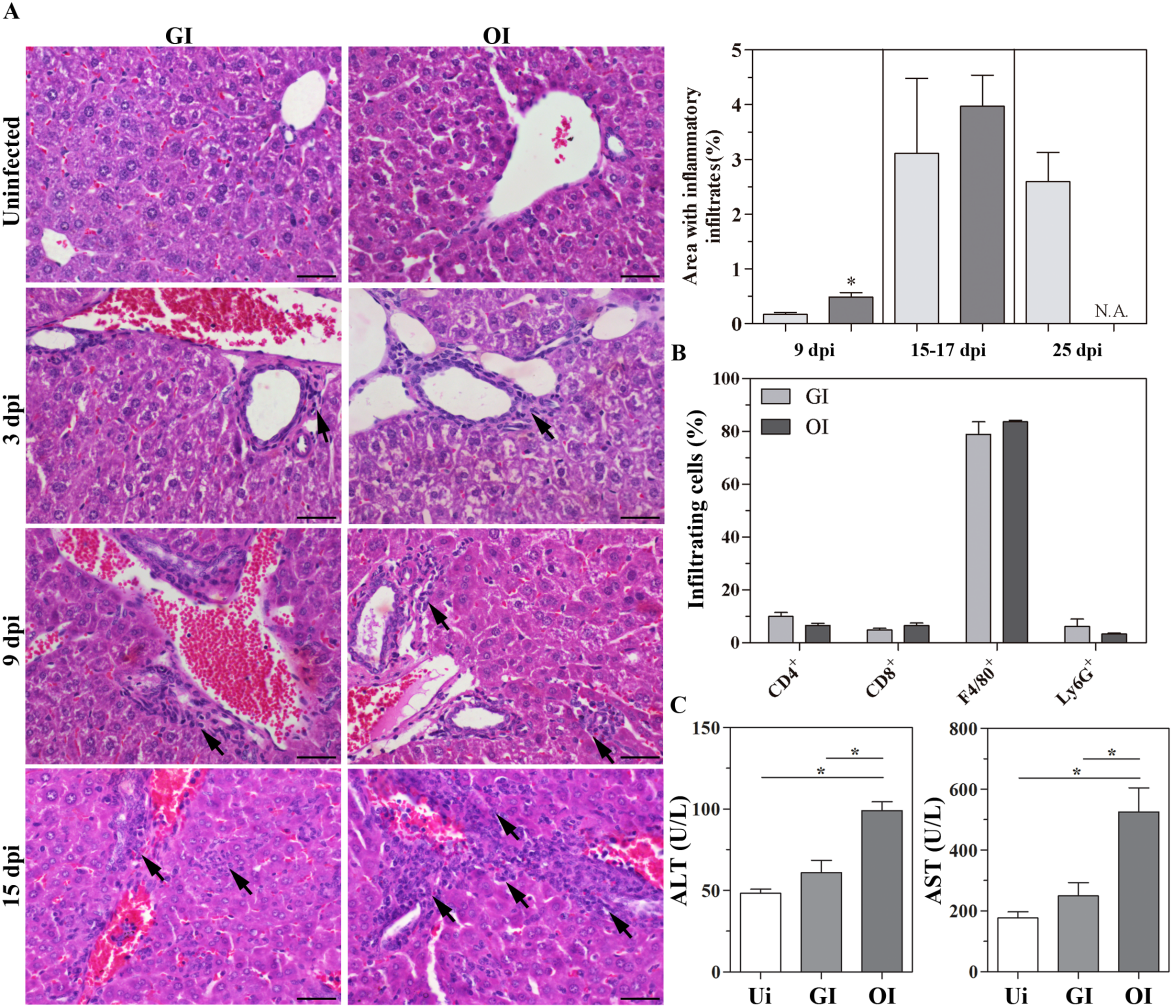
Liver histology during acute *Trypanosoma cruzi* infection after GI and OI inoculation. Male BALB/c mice were infected with 5x10^4^ tissue culture-derived trypomastigotes forms of *T*. *cruzi* (Tulahuén strain) through gavage (GI) or within oral cavity (OI). A) Hematoxylin and Eosin stained sections demonstrating liver histological lesions in terms of inflammatory foci. For the quantification of inflammatory infiltrate, the relative area of infiltration from 25 fields (200X) was analyzed. Pictures represents cells-rich infiltrated areas. n: GI, 9 dpi = 4, 15–17 dpi = 4, 25 dpi = 5; OI, 9 dpi = 4, 15–17 dpi = 9. B) Immunofluorescence analyses demonstrating the percentage of each subset present within the tissue after 16/17 dpi, CD4^+^, CD8^+^, F4/80^+^ and Ly6G^+^ cells. Numbers represent mean± SEM. n = 3 mice/group (two different section from each mouse). C) ALT and AST activity (17 dpi) in sera. All statistical analyses were performed using one-tailed Mann-Whitney test, GraphPad Prism 5. Comparison between GI and OI groups, and each one of them with uninfected mice. *, p = 0.05; **, p = 0.01; ***, p = 0.001. Bars represent 20 μm. Arrows show inflammatory infiltrates.

Orally administered drugs/antigens are usually absorbed by the gastro-intestinal tract and transported to the lymphatic or hepatic portal system [30]. Moreover, the liver is known to be a target tissue for the parasite and plays a role in clearance of blood trypomastigotes [31]. As such, the liver may be involved with acute phase development in an orally infected host. To test this hypothesis, a comparative analysis of hepatic sections between **GI** and **OI** infected mice was necessary. As judged by liver histopathological analysis in 3, 9, 15–17, 25 dpi, **OI** promoted severe hepatitis. During the initial stages of infection (3–9 dpi), hepatic infiltrates showed mild intensity mainly around the small and medium size vessels and it was higher in **OI** than **GI**. As the infection develops (15–17 dpi), infiltration notably increased also affecting the parenchyma in both **OI** and **GI** mice (Fig 3A and S4 Table). Amastigote nests were rarely detected in the liver. Moreover, it was evident that medium vessels with blood stasis and suggestive formation of thrombotic masses occurred mainly in the **OI**-infected mice (S4 Table). Picrossirius Red staining revealed progressive deposits of collagen in blood vessel walls, mainly in **OI** infected mice (S4 Table). Immunofluorescence analysis from two different lobes showed that the inflammation was mainly composed by F4/80^+^ macrophages. However, CD4^+^ cells, CD8^+^ cells and Ly6G^+^ neutrophils were also observed (Fig 3B). Furthermore, the **OI** group presented hepatic damage given the increased ALT and AST serum levels (17 dpi). Apoptotic (TUNEL^+^) cells were also detected in the inflammatory infiltrate and at the parenchyma at 16 dpi (Figs 3C and S4).

### The pattern of cytokine secretion is distinct between OI and GI infected mice

**Fig 4:**
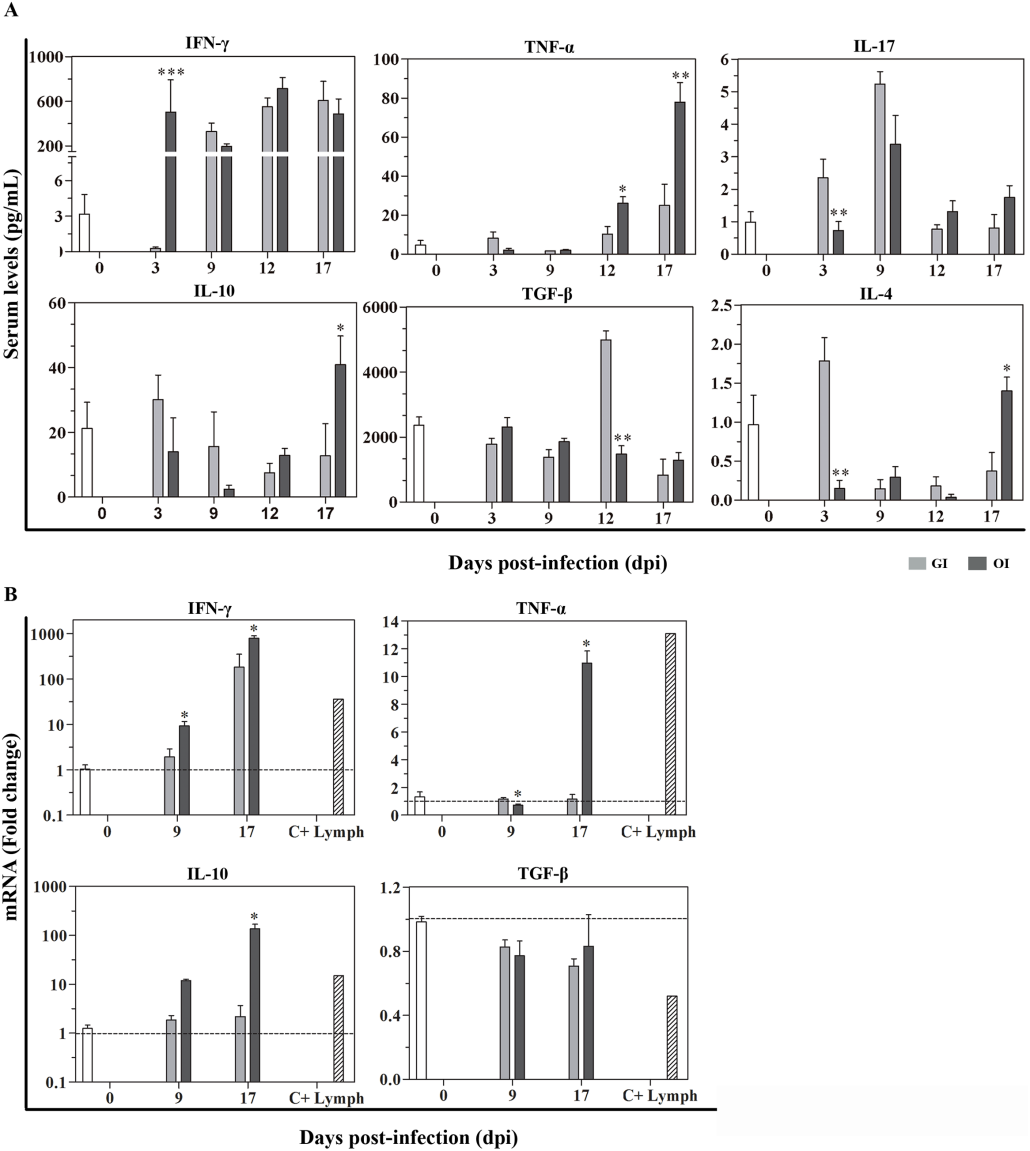
Cytokine production in GI and OI infected mice. Male BALB/c mice were infected with 5x10^4^ tissue culture-derived trypomastigotes forms of *T*. *cruzi* (Tulahuén strain) through gavage (GI) or within oral cavity (OI). A) In the course of acute infection, serum was isolated and levels of cytokines (IFN-γ, TNF, IL-17, IL-10 and TGF-β) were quantified in uninfected control and infected mice by a multiplex analysis. The results are expressed as the mean values (±SEM) for each group/day post-infection. n: IFN-γ, uninfected (0) = 12; 3 dpi GI = 11, OI = 5; 9 dpi GI = 8, OI = 5; 12 dpi GI = 9, OI = 4; 17 dpi GI = 4, OI = 6. TNF, uninfected (0) = 11; 3 dpi GI = 10, OI = 10; 9, 12 dpi, GI = 3, OI = 3; 17 dpi, GI = 6, OI = 11. IL-17, uninfected (0) = 12; 3 dpi, GI = 10, OI = 10; 9 dpi, GI = 3, OI = 3; 12 dpi, GI = 5, OI = 5; 17 dpi, GI = 6, OI = 14. TGF-β, uninfected (0) = 6; 3 dpi, GI = 4, OI = 4; 9 dpi, GI = 5, OI = 5; 12 dpi, GI = 5, OI = 4; 17 dpi, GI = 2, OI = 5. IL-10 and IL-4, uninfected (0) = 6; 3, 9, 12 dpi, GI = 6, OI = 6; 17 dpi, GI = 3, OI = 8. B) Cytokine gene expression levels were performed by RT-qPCR. Total RNA was isolated from the heart at different days post-infection, and the reaction was performed using SYBR Green Master Mix. HPRT and β-actin were used as housekeeping genes. RT-qPCR data were normalized to the housekeeping genes, and fold changes were determined compared with uninfected controls, using the Expression Suite software. Lymphocytes from subcutaneous lymph nodes of infected mice were stimulated with anti-CD3 and used as positive control of cytokines production (C+ Lymph). Statistical analysis was performed using ΔCt values. n: uninfected (0) = 5–9; 9 dpi GI = 3, OI = 2–3; 17 dpi GI = 2–3, OI = 2–3. Both sets of data were analyzed using one-tailed Mann-Whitney test, GraphPad Prism 5. *, p = 0.05; **, p = 0.01; ***, p = 0.001. *GI versus OI. Kruskal-Wallis (Dunn’s post-test) for group kinetics: A) IFN-γ, TNF and IL-10 increased in both groups compared with uninfected. IL-17 presented a non-significant increase followed by decrease. TGF-β was only elevated in GI group at 12 dpi in sera. B) IFN-γ increased in both groups compared with uninfected. TNF and IL-10 increased in OI whereas TGF-β decreased in GI.

In immune response to infection, Th1, Th2, Th17 and regulatory cytokines play an important role in the control of parasite and disease development [32]. To investigate the impact of the route of infection on systemic cytokine levels, a thorough multiplex analysis was performed. As demonstrated in Fig 4, **OI** mice showed higher type 1 cytokines levels, i.e., IFN-γ (3 dpi) and TNF (12, 17 dpi) but also IL-10 (17dpi), than **GI** mice. Conversely, IL-17 (3 dpi) and the regulatory cytokine TGF-β (12 dpi) was increased in **GI** mice (Fig 4). Elevated levels of pro-inflammatory cytokines are also associated with cardiac tissue damage [32]. In order to analyze cytokine presence in the cardiac tissue of infected mice, real time PCR was performed for IFN-γ, TNF, IL-10 and TGF-β cytokines. Interestingly, IFN-γ, TNF, and IL-10 gene expression was increased in the **OI** group (Fig 4). Moreover, TNF increase was evident in **OI** mice, but not **GI** mice (Fig 4B).

### TNF production in the heart and liver

**Fig 5:**
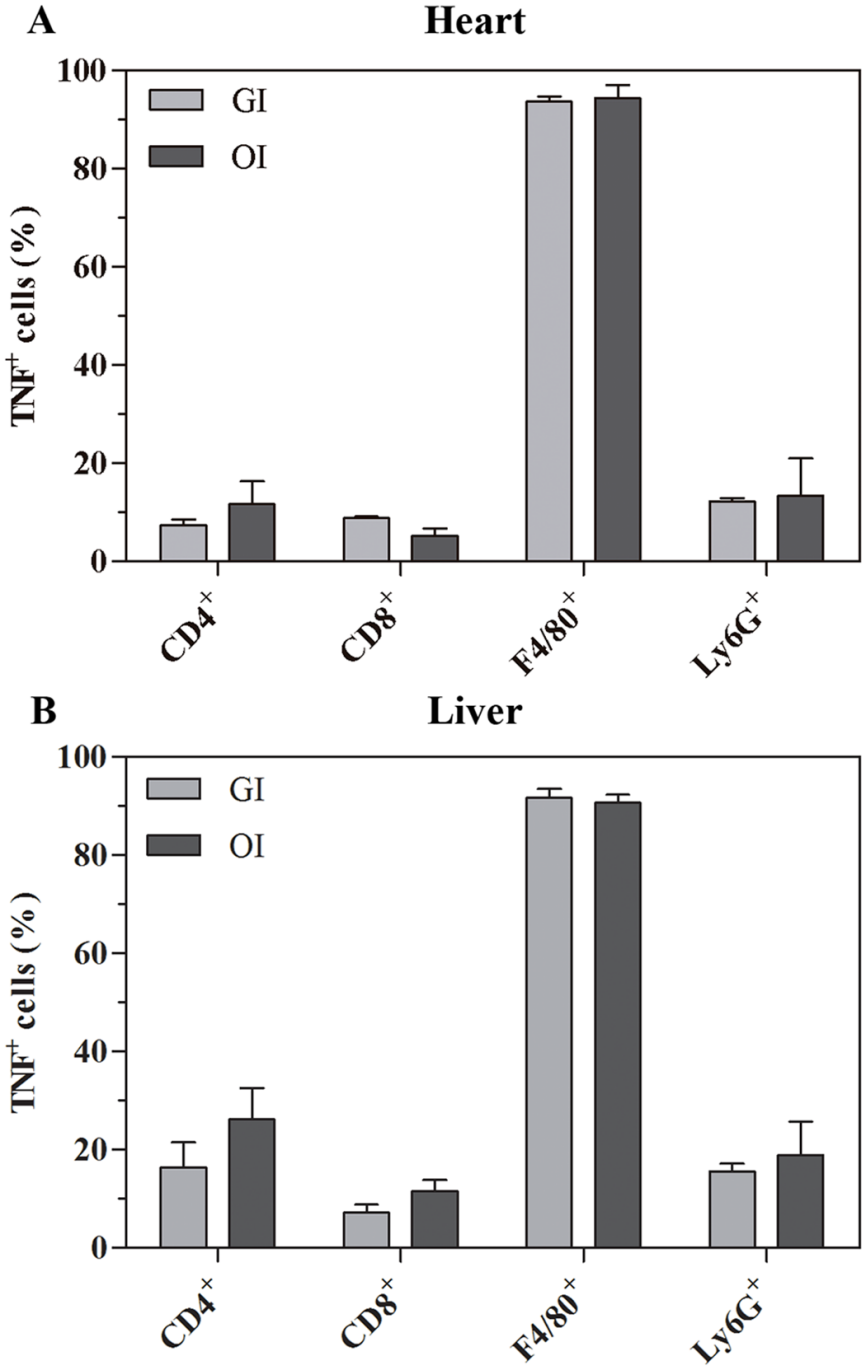
F4/80^+^ cells are the major TNF-producing cells. Male BALB/c mice were infected with 5x10^4^ tissue culture-derived trypomastigotes forms of *T*. *cruzi* (Tulahuén strain) through gavage (GI) or within oral cavity (OI). Immunofluorescence analyses demonstrated the percentage of TNF-producing cells among each subset present within the tissue after 16/17 dpi, CD4^+^, CD8^+^, F4/80^+^ and Ly6G^+^ cells. A) heart and B) liver. Numbers represent mean±SEM. n = 3 mice/group (two different sections from each mouse).

Immunofluorescent staining from heart and liver samples of 16 dpi mice showed the presence of TNF in these tissues. TNF labeling was evident in inflammatory cells, mainly in macrophages (Figs 5 and S2). **OI** hepatic sections presented a higher number of macrophages than **GI** (mean ± SEM, standard error of the mean: 1200 ±18.68 *versus* 963 ± 43.15, respectively) analyzed in 14 fields from two different liver lobes.

### Elevated TNF production is involved in orally infected mice mortality

**Fig 6:**
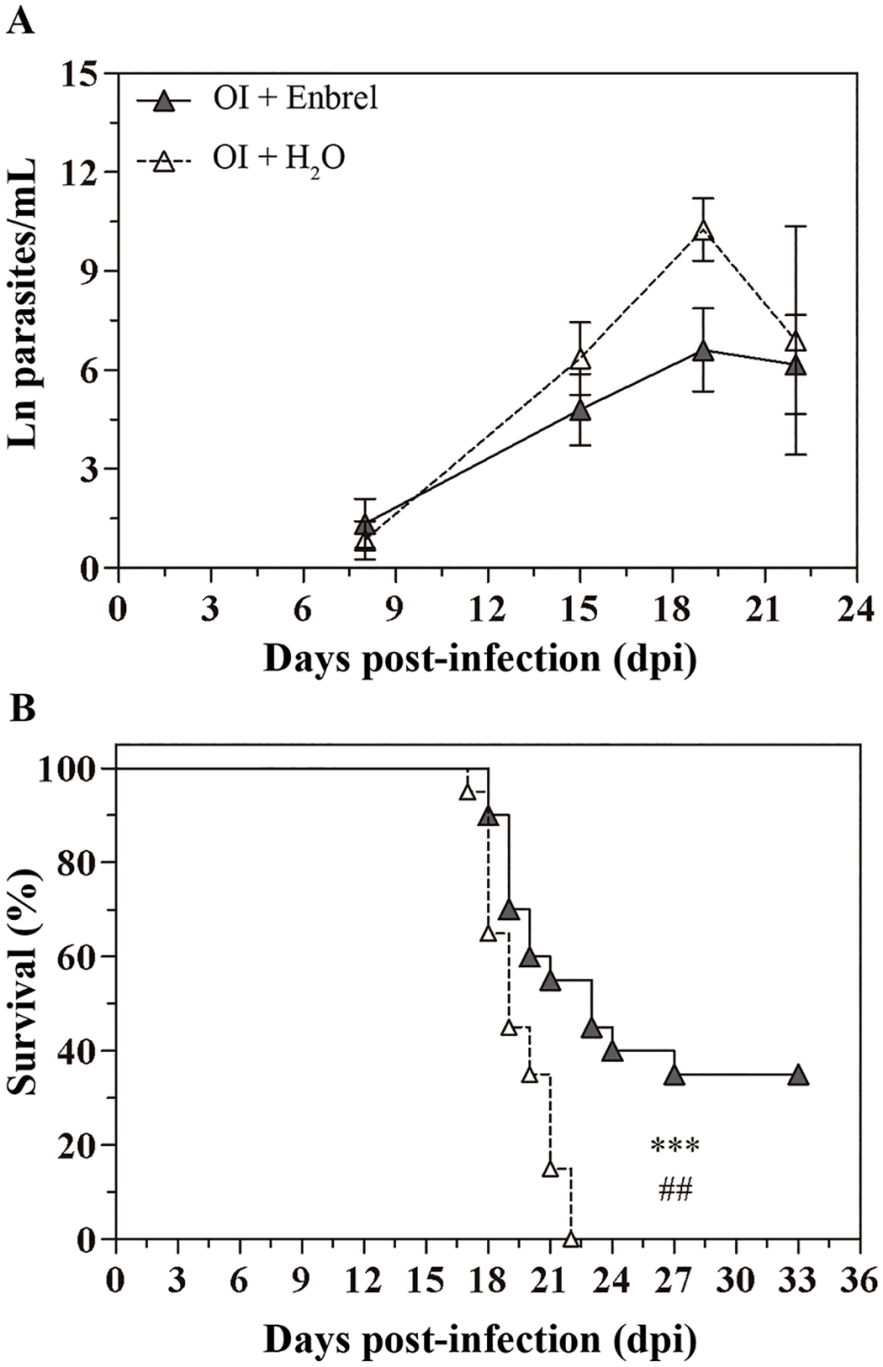
Anti-TNF therapy improves the survival of orally infected mice. Male BALB/c mice were infected with 5x10^4^ tissue culture-derived trypomastigotes forms of *T*. *cruzi* (Tulahuén strain) within oral cavity (OI). Anti-TNF treatment with etanercept began after 14 dpi and was performed weekly. A) Parasitemia (mean and SEM of ln parasite/mL) and B) mortality were followed during the acute phase and subjected to statistical analysis. Parasites were counted by light microscopy, and parasitemia calculated by Brener method. Statistical analysis was performed using GraphPad Prism 5. For parasitemia comparisons on days 8, 15, 19 and 22 dpi, one-tailed Mann-Whitney test was used. n: OI+enbrel = 14–23, OI+H_2_O = 3–23. Survival was analyzed by Log-rank (Mantel-Cox) (***) and Gehan-Breslow-Wilcoxon (##) tests. n = 20 mice (equivalent to 100%). * p = 0.05; ** p = 0.01; *** p = 0.001.

To evaluate the impact of elevated TNF serum levels in host resistance, **OI** mice were treated with the anti-TNF, etanercept (Enbrel). Enbrel treatment in **OI** mice started at 14 dpi. As demonstrated in Fig 6, our protocol of TNF blockade did not affect blood trypomastigotes number, but treated mice presented a longer survival than non-treated ones (Fig 6).

## Discussion

Currently, oral transmission of Chagas disease is the most important route of transmission in Brazil (70–80% of cases) [7]. Venezuela, Colombia, Bolivia, Argentina and Ecuador have also reported to have acute cases of Chagas disease associated with food/beverage consumption, but a significant study in the region is lacking [5]. These orally infected patients progress with a highly symptomatic disease (fever, facial edema, exanthema, hemorrhage, meningoencephalitis, abdominal pain, others), beyond the classical cardiac involvement. Additionally, increased mortality rate is marked in the first 2 weeks (8–35%), surpassing the calculated mortality produced by the disease resulting from the biting of infected insect vectors (5–10%) [18].

It has been well accepted that the route of parasite entry into the host is a key factor in Chagas pathogenesis [21]. Previous reports demonstrated that systemic *versus* mucosal infection promotes a distinct disease pattern. It has been shown that CFI mice infected with the Peruvian strain (TcII) of *T*. *cruzi* through systemic routes **IP**, intravenous, or subcutaneous (s.c) have higher infection rates (67–100%) and mortality than mucosal routes [**OI**, **GI**, intrarectal, genitalia, or conjunctival] (17–67%) [33]. Moreover, Caradonna and Pereiraperrin [34] infected BALB/c and C57BL/6 mice with the Tulahuén strain (TcVI) of *T*. *cruzi* via s.c. and intranasal routes (i.n.), and found higher mortality in the s.c. group. Furthermore, mice infected via the i.n. route developed a higher brain parasitism and lower parasitemia than animals infected via the s.c. route, suggesting a preferential homing of the parasite to the brain after intranasal administration [34]. Interestingly, when mice are infected with the same strain simulating natural infection, by oral (oropharynx) or cutaneous (over a puncture wound) challenge, insect-derived trypomastigotes are more infective through oral inoculation [19]. Regardless of DTU (TcI or TcII strains), infection through gavage (intragastrically) presents less infectivity, parasitemia and mortality than intraperitoneal injection [35], as we have also observed with the Tulahuén strain (TcVI). Here, we demonstrated that **OI** infected mice induced a higher infective rate when compared with **GI** infected mice (S1 Fig). As well as the inoculation route, certain factors, such as the inoculum size, DTU and *T*. *cruzi* developmental stage, may be involved in the disease outcome. Previous studies demonstrated that the Y strain (TcII) with 5x10^4^ blood trypomastigotes infection by gavage (**GI**) showed higher parasitemia than the Colombian (TcI) strain [35]. In addition, **IP** infection with blood trypomastigotes shows higher infectivity than insect-derived trypomastigotes [36].

Gp82 and gp30 are involved in gastric invasion and can be differently expressed among distinct strains and developmental stages. Culture-derived metacyclic trypomastigotes from the Tulahuén strain (TcVI) was already described as expressing gp82 and CL strain, from the same DTU, presented high expression of gp82 and gp30, similarly to human isolates. These strains induce high parasitemia [25,27,37,38]. Another glycoprotein, gp90 (impairs cell invasion), is less expressed in CL (TcVI) parasites, when compared with the SC strain (isolated from a patient who ingested contaminated sugar cane). However, the SC strain is highly infective because its gp90 is susceptible to gastric juice [27].

Cortez and colleagues identified the mucin-ligand sequence present in gp82 from metacyclic Y strain (TcII) and its counterparts Tc85-11, involved in cell invasion by tissue culture-derived trypomastigotes (TCT) [39]. In our study, both TCT and insect derived trypomastigotes were able to promote mice infection trough the **GI** route. Moreover, it has been shown that both insect-derived metacyclic trypomastigotes (triatomine insects that were crushed along with fruits) and blood/ cell-derived trypomastigotes (consumption of infected wild *T*. *cruzi* reservoir hosts- marsupials, bats and others) are associated with human outbreaks of oral Chagas disease [5,40,41].

The primary site of parasite entry in oral infection is still unknown. Previous data proved that in orally infected mice, parasites are not detected within the oropharynx and esophagus, instead, amastigote nests are present in the stomach [23]. In line with these findings, another group suggested that parasite glycoproteins, such as gp82 and gp30, are involved in gastric invasion following intragastric/intrapharyngeal inoculation [27,37,42,43]. Altogether, these reports name intragastric, intrapharyngeal and oral cavity parasite delivery as “oral” infection. Here, we demonstrate that BALB/c mice infected through the oral cavity (**OI**) experienced a higher infective rate when compared with **GI** infected mice. To our knowledge, this constitutes the first report addressing the potential differences in disease outcome according to **OI** or **GI** route. After reaching systemic circulation, *T*. *cruzi* can multiply inside several cell types, such as macrophages, fibroblasts, skeletal and cardiac muscle, neurons and epithelial cells. Notably, the parasite presents tropism for cardiac tissue, where it forms amastigote nests and triggers immune cell recruitment [32]. Here, we demonstrated that in spite of the lower parasitemia and mortality, **GI**- mice developed a more severe myocarditis than **OI** mice, suggesting that cardiac involvement might not be related to the elevated mortality of the **OI** group. Tulahuén (TcVI) is not described as myotropic strain, but, this reticulotropic strain still affects the heart of infected mice. Moreover, in our study, this strain induced differences in inflammatory infiltration in the heart and damages in the liver between **OI** and **GI** groups. In addition, the intragastric infection with Colombiana (TcI) myotropic strain induces inflammation and amastigote nests formation in the heart [24].

It has been shown that *T*. *cruzi* is able to infect the reticuloendothelial system, including bone marrow, spleen and liver. Moreover, **IP** infection leads to apoptosis of hepatic cells and liver inflammation due to TNF production. In this regard, it was previously shown that the Tulahuén strain of *T*. *cruzi* induces TNF- production and death of hepatocytes by apoptosis, involving tBid and Bax proteins and influencing organ function [44]. **OI** and **GI** infection also promote apoptosis in the liver and in the heart of acute infected mice (S4 Fig). Interestingly, hepatic damage in **OI** is severe, as judged by histopathology and elevated ALT and AST serum levels. It has been shown that hepatocytes are not commonly infected *in vivo*, but amastigote nests may be observed in sinusoidal and Kupffer cells [45]. Here, we have hardly detected amastigote in hepatic tissue, most likely in Kupffer cells. This scarcity of *T*. *cruzi* amastigotes in hepatic cells is assumed to occur because of efficient control of the parasite clearance within this organ [46]. Additionally, the liver is described as the first line of protection against pathogens and in tolerogenic responses to antigens coming from the gut through the portal system [46,47,48,49]. Our results demonstrated that macrophages in the liver are TNF^+^ cells (Fig 5). Activated macrophages producing TNF in the liver may be involved with *T*. *cruzi* killing in the tissue. Nevertheless, considering the role of TNF in cell death, apoptotic bodies were also evident in TNF-rich regions (S4 Fig).

Paralleling parasitological and histological differences, **GI**- and **OI**- mice presented elevated IFN-γ levels in the serum, whereas higher levels of TNF were only observed in **OI** mice. Cellular adaptive immune response during infection is essential for parasitism control [50]. Cytokines play important roles in regulating *T*. *cruzi* replication and the immune response of infected animals. Th1 cytokines, such as IFN-γ and TNF, are involved in parasite control and host resistance whereas Th2 cytokines, such as IL-4 and IL-5, are associated with host susceptibility [32]. In the initial stage of infection, parasite DNA and surface glycoconjugates are able to trigger innate immune response through TLR-2, -4 and -9 in macrophages and dendritic cells, enhancing their endocytic capacity and killing by oxidative burst. Pro-inflammatory cytokines, such as IFN-γ, favors inflammatory cell activation that migrate to control parasite burden [50]. Elevated TNF levels, as observed in **OI**, may be associated with cardiac, spleen, hepatic damage, and toxic shock in mice, as reported in other studies [51,52]. Reinforcing this view, **OI** mice showed a peak in TNF serum levels at 17 dpi, the time when they started to die. **GI** mortality also started at this time point, but the rate was lower than in **OI** mice. In the same sense, TNF detection by RT-PCR was higher in cardiac cells from **OI** than **GI** mice. Extending these findings, we have also shown that both **GI** and **OI** mice presented a high serum concentration of IL-10 and IL-4 (17 dpi). Meanwhile, the **OI** group had lesser amounts of the TGF-β regulatory cytokine. Both cytokines were proven to inhibit macrophage microbicidal function and protect the host from tissue damage [52,53]. It has been shown that IL-17 producing cells contribute to the formation of the gastrointestinal barrier [54]. Our results demonstrated that, as expected, parasite inoculation into mucosal routes (**GI** and **OI**) triggered IL-17-producing cell activation, given its high serum levels.

Studies addressing different routes of infection are relevant as they seem to lead different immune responses and disease outcome. Infection with bacteria, such as *Listeria monocytogenes*, *Streptococcus pyogenes* and *Francisella tularensis* through mucosal sites (intranasal or oral) promotes Th17 response with the systemic route (intravenous/s.c.) triggering a Th1 response. Antigens delivered into mucosal tissues stimulate these IL-17 producing cells [55], which was also observed in *T*. *cruzi* infection.

In **OI** animals, elevated circulating levels of TNF after 17 dpi were strongly associated with hepatic damage and death. Similar results were observed in mice deficient of IL-10, which display enhanced hepatic cell destruction and toxic shock by increased TNF. To comprobate that TNF is involved in the death of **OI** mice, we blocked this molecule. Because TNF is critical to control parasitemia [32,48], etanercept administration started at 14 days post-infection, at the time that parasitemia could also be controlled by humoral response. Etanercept treatment statistically delayed mortality without altering the levels of parasitemia, revealing the critical role of TNF in the course of **OI** infection, (Fig 6). Strikingly, similar results were also observed by Rodriguez-Angulo [56].

In fact, there is a clear association between peaked 17 dpi TNF circulating levels and liver alterations in terms of inflammatory infiltrates, which may impact organ functionality.

For several years, the **IP** route of *T*. *cruzi* infection has been chosen as the main pathway of parasite challenge in experimentally infected hosts in attempting to reproduce the vectorial route. We and others have already demonstrated that the route of parasite challenge is a major issue in terms of infection outcome and hence, worth considering in the human counterpart, mainly because oral transmission is becoming more epidemiologically relevant. Here, we clearly demonstrate that the host response differs when parasites are delivered into the mouth or by gavage. If compared with patients, oral outbreaks are related to contaminated food ingestion [5] and interestingly, the appearance of facial edema is frequent in these patients [57]. Parasite/antigens can be captured in the oral mucosa by tolerogenic dendritic cells that produce IL-10 and IL-12 (regulatory and inflammatory profile), or in the gastrointestinal tract from where they are drained to the liver by the portal system [49,58]. Even considering oral infection, it should not be assumed that the infectious processes are the same when parasites are delivered into the oral cavity or by gavage (intrapharyngeal /gastrointestinal).

On a theoretical basis, our studies also raise a series of questions worth exploring. For instance, the immunopathological and parasitological consequences of oral and systemic infection in the same host at different times are of interest. Whether there is a favorable influence of oral or systemic infection depending on which comes first is also interesting. From a translational standpoint, the finding that the route of parasite contact is involved in a differential pathophysiology and disease morbidity provides information that sounds suitable for clinical management and disease control strategies.

## Materials and Methods

### Animals and infection

Male BALB/c mice were obtained from the Oswaldo Cruz Foundation animal facilities (Brazil). Mice (6–8 weeks old) were infected by gavage as a gastrointestinal attempt (**GI**) or in the oral cavity (**OI**) with 5x10^4^ *T*. *cruzi* tissue culture-derived trypomastigotes forms (Tulahuén strain, TcVI [59]). Parasites were obtained from infected cultures of a highly susceptible lineage of monkey kidney epithelial cell line (Vero cells) [60]. Oral Chagas disease outbreaks are related to the consumption of contaminated food with infected triatomine excreta (metacyclic trypomastigotes) or consumption of wild *T*. *cruzi* reservoir hosts (blood and cell-derived trypomastigotes). The purpose of this study was to analyze the immune response of infected mice. As components from excreta could interfere in this response, some experiments were performed with trypomastigotes mixed with or without non-infected *Triatoma infestans* excreta. In these infections, uninfected mice were stimulated with saline. For both **GI** and **OI**, mice were maintained starving 4 hours before and at least 15 minutes after inoculation (100 μL of parasites suspension). **GI** was performed using a gavage canule and **OI**, by pipeting the volume into the mouth.

As **GI** infection is the less effective route of infection, 5x10^4^, 10^5^ or 10^6^ inocula size were tested by gavage. In this study, the lower (5x10^4^) inoculum capable of infecting mice **GI** was chosen.

For comparative purposes, in some experiments, intraperitoneally infected mice were also analyzed.

In other series of experimental rounds, mice were also infected concomitantly with an inoculation of antacid (19.4 mg/Kg of Magnesium Hydroxide [Mg(OH)_2_] suspension, Phillips- Brazil), and trypomastigotes.

### Ethics statement

This study was performed in strict accordance with the recommendations in the Guide for the Care and Use of Laboratory Animals of the Brazilian National Council of Animal Experimentation and the Federal Law 11.794 (10/2008). The Institutional Ethics Committee for Animal Research of the Oswaldo Cruz Foundation (CEUA-FIOCRUZ, License: LW-23/12) approved all of the procedures used in this study.

### Parasitemia and survival

Parasitemia was detected at different days post-infection (dpi) by counting trypomastigotes in 5 μL of tail blood, and parasite number was calculated using the Brener method. Mortality and survival were followed until 60 days post-infection.

Parasitemia and survival rates were evaluated with all inoculated animals. Due to differences in infectivity from **OI** and **GI** mice, in the other figures throughout this paper were included infected mice with patent parasitemia.

### Heart and liver histopathological analyses

Hearts and livers from infected or uninfected mice were fixed in buffered 5% formalin. Heart samples were sliced longitudinally in two parts, and the liver, in several fragments from different lobes. Paraffin-embedded 5 μm sections were mounted on glass slides and stained with the Hematoxylin-Eosin and Picrossirius Red technique to evaluate infiltrating cells and collagen fibers. Photos were taken using the Leica DM 2500 microscope and then analyzed using Image J software. In the heart, the percentage area with inflammatory infiltrates or amastigote nests was calculated by analyzing 50 fields/section/mouse. In addition to the area of inflammation, the degree of pericarditis and myocarditis was classified according to the extension of infiltrating area: +, very mild (similar with uninfected); ++, mild (small areas of infiltrates); +++, moderate (moderate areas of infiltrates); ++++, severe (extensive areas of infiltrates); +++++, very severe (very extensive areas of infiltrates).

In hepatic tissue, the percentage area with inflammatory infiltrates was calculated by analyzing 25 fields/mouse (two different sections from each mouse). The inflammatory infiltrates were scored as:-, without infiltrates (without alterations); +, mild lesions areas (small focal infiltrates mainly around the vessels, parenchyma not infiltrated); ++, moderate areas of infiltrates (infiltrates with intermediate size, around the vessels, but few diffuse and microgranulomas also within the parenchyma and thrombus formation in some vessels); +++, severe areas of infiltrates (areas of infiltrates with microgranulomatous structure, diffuse infiltrates within the parenchyma and more vessels with thrombus formation), ++++ very severe (extensive areas of infiltrates with microgranulomatous structure and diffuse infiltrates within the parenchyma).

### Serum cytokine analysis

Mice were bled by cardiac puncture at 3, 9, 12, 15, 17, 21, 25 and 27 days post-infection. Serum was stored frozen at -70°C until used. Serum levels of IL-4, IL-10, IL-17, IFN-γ, TNF and TGF-β were measured in a Multiplex analysis, Milliplex MAP—mouse cytokine / chemokine magnetic bead panel kit and TGFβ1 single plex kit (Merk Millipore, USA). The assay was performed by the Gênese Institute of Clinical Analyses, São Paulo/SP, Brazil. Cytometric beads assay (CBA), using the Mouse Th1/Th2/Th17 Cytokine kit (BD Biosciences, USA) was also performed for serum cytokine analysis according to the manufacturer’s instructions. Samples were immediately acquired using FACSCanto II (Becton and Dickinson, USA) equipped with FACSDiva software (Becton and Dickinson, USA). Data were analyzed using FCAP Array software (Becton and Dickinson, USA).

### Cytokine gene expression in the heart

For real-time quantitative RT-PCR (RT-qPCR), the total RNA from one-half of the heart (longitudinal section; average weight: 87.9 mg) samples was extracted using Trizol Reagent (Ambion, Life Technologies) associated with the RNeasy Mini kit assay (Qiagen), from the phenol-chloroform aqueous phase, following the manufacturer’s instructions. Reverse transcriptase reactions were performed on 3.5 μg RNA using Super Script II kit (Invitrogen, USA) according to the manufacturer’s instructions. Real-time RT-PCR assays were performed on StepOnePlus (Applied Biosystems, USA) using Power SYBR Green Master Mix (Applied Biosystems), and the primers for cytokines, IFN-γ, TNF, TGF-β, IL-10 and IL-17, purchased from IDT (Integrated DNA Technologies) (S2 Table). Hypoxantine-guanine phosphoribosyltranseferase (HPRT) and β-actin genes were used as endogenous housekeeping controls (S2 Table). cDNA were diluted 1:10 and reactions were performed in duplicate using 2 μL per reaction, in a total volume of 20 μL. After amplification, dissociation curves were performed, revealing only one melting peak for each amplified fragments. Relative quantifications of target gene levels were performed using ΔΔCt method [61]. RT-qPCR data were normalized to the housekeeping genes, and fold changes were determined compared with uninfected control samples using the Expression Suite software (Life Technologies, USA). Statistical analysis was performed from ΔCt values.

### ALT and AST measurement

Blood samples obtained from mice of all groups were allowed to coagulate, and the serum was then isolated. Serum **ALT** (alanine transaminase) and **AST** (aspartate transaminase) activities were measured with the Reflotron (Roche, Germany) apparatus according to the manufacturer's instructions.

### Immunofluorescence analysis

Hearts and livers from infected or uninfected mice were included in tissue tek (OCT, Sakura, USA). Heart samples were sliced longitudinally in two parts, and the liver, in several fragments. To evaluate TNF-producing cells, double immunofluorescences were performed. Cryosections with 3 μm were fixed in acetone for 5 minutes at 4°C. After two washes in cold PBS, a blocking solution of 10% normal serum goat and 1% BSA was applied to the sections for 1 hour at room temperature. Samples were incubated overnight at 4°C with primary antibodies and washed three times in PBS and subjected to the appropriate secondary antibodies for 45 minutes at room temperature, Alexa-488 goat anti-rat for anti-CD4, -CD8, -F4/80 and -Ly6G, and Alexa-546 goat anti-rabbit for anti-TNF. The characteristics of the antibodies used in immunostaining are listed in S1 Table. After three washes in PBS, the slides were mounted in ProLong Gold Antifade Mountant with DAPI (Molecular Probes, USA).

To evaluate apoptosis, the ApopTag *In Situ* Apoptosis Detection Kit (Merk Millipore, USA) was used following the manufacturer instructions. Counterstaining/mounting was performed using ProLong Gold Antifade Mountant with DAPI (Molecular Probes, USA).

All images were visualized using the Zeiss microscope (Germany) and digitalized using AxioCam HRm and AxioVision Rel 4.8 software.

The subsets (CD4^+^, CD8^+^, F4/80^+^ and Ly6G^+^) present in the infiltration were counted in 10 and 14 fields/photos for the heart and liver, respectively, and TNF^+^ cells were quantified. Two sections from the heart and two from different liver lobes from each mouse were analyzed. The percentage of CD4, CD8, macrophages and neutrophils was calculated over the sum of all subsets from different sections.

### Anti-TNF treatment

Orally infected BALB/c mice were treated intraperitoneally with a quimeric anti-TNF protein (Etanercept Enbrel, Wyeth Pharmaceuticals, 0.83 mg/Kg). The treatment began at the 14th day post-infection with weekly subsequent doses. Parasitemia and mortality were analyzed throughout the course of infection.

### Statistical analysis

Kruskal-Wallis (Dunn’s post-test) or Mann-Whitney tests were used for the statistical analyses. Survival was analyzed by Log-rank (Mantel-Cox Test) and Gehan-Breslow-Wilcoxon test. P *values* < 0.05 were considered statistically significant. Tests were performed using GraphPad Prism 5.

### Accession number for the genes and proteins mentioned

Genes: IFN-γ (NM_008337.3), TGF-β (gb_M13177.1), TNF (NM_013693.3), IL-17A (NM_010552.3), IL-10 (NM_010548.2), HPRT (gb_J00423.1), β-actin (NM_007393.3). Proteins: IFN-γ (gb_EDL24379.1), TGF-β1 (NP_035707.1), TNF (gb_AAC82484.1), IL-17A (NP_034682.1), IL-10 (NP_034678.1), gp82 (gb_ABR19835.1), gp30 (gb_AEF13371.1), gp90 (gb_AAM47176.1|AF426132_1), mucin (gb_AAA39755.1), IL-4 (NP_067258.1), TLR-2 (gb_AAD49335.1|AF165189_1), TLR-4 (NP_067272.1), TLR-9 (NP_112455.2).

## Supporting Information

S1 Fig *Trypanosoma cruzi* infection presents differences in infectivity according to the inoculation route. Male BALB/c mice were infected with 5x10^4^ tissue culture-derived trypomastigotes through intraperitoneal (IP), gavage (GI) or oral (OI) inoculation. Infectivity was obtained from the percentage of mice presenting parasitemia over the total number of mice inoculated with parasites. Kruskal-Wallis (Dunn’s post-test) was performed and symbols represent comparison to IP or OI, * and #, respectively. Statistical analysis was performed using GraphPad Prism 5. **^/##^ p = 0.01
(TIF)

S2 Fig F4/80^+^ macrophages are the main TNF producer cells. Male BALB/c mice were infected with 5x10^4^ tissue culture-derived trypomastigotes through gavage (GI) or oral (OI) inoculation. A, heart and B, liver tissue cryosections were submitted to double immunostaining for F4/80 (green) and TNF (red). n = 3 mice/group (two sections from each). Bars represent 100 μm.
(TIF)

S3 Fig Mild collagen production in heart and liver from infected mice. Male BALB/c mice were infected with 5x10^4^ tissue culture-derived trypomastigotes through gavage (GI) or oral (OI) inoculation. Paraffin-embedded sections were stained with Picrossirius to reveal collagen production. A, heart, counterstained with Hematoxylin and B, liver. n = 3–5 mice/group/dpi. Bars represent 50 μm.
(TIF)

S4 Fig Apoptosis in heart and liver. Male BALB/c mice were infected with 5x10^4^ tissue culture-derived trypomastigotes through gavage (GI) or oral (OI) inoculation. TUNEL technique was applied to A, heart and B, liver cryosections for apoptosis detection. C, TNF staining (red) associated with structures that were also related to TUNEL staining (red) within the liver from infected mice. n = 3 mice/group (two sections from each). Bars represent 100 μm.
(TIF)

S1 Table Antibodies applied in immunofluorescence. Abbreviations: Ig, immunoglobulin; mAb, monoclonal antibody.
(DOCX)

S2 Table Primers sequences for target mRNAs. Sequences of each primer, manufacture and used concentration are indicated. Holding stages were at 95°C for 10 minutes for all primers, and cycling stages (40x) varied depending on the target. For IL-10, IFN-γ, TGF-β cycling stage was at 95°C for 15 seconds and at 63°C for 1 minute. For TNF, at 95°C for 15 seconds and 64°C for 30 seconds. HPRT and β-actin cycles were performed in the same conditions of each target.
(DOC)

S3 Table Heart histopathological analysis. Male BALB/c mice were infected with 5x10^4^ tissue culture-derived trypomastigotes forms of *T*. *cruzi* (Tulahuén strain) through gavage (GI) or oral cavity (OI). Hearts were harvested at different days post-infection (dpi), fixed and embedded in paraffin. Histological longitudinal sections were stained by Hematoxylin-Eosin and Picrossirius Red. The table represents degree of pericarditis and myocarditis was classified as: +, very mild; ++, mild; +++, moderate; ++++, severe; +++++, very severe. Amastigotes nests were observed inside or next infiltrating areas. Infected mice presented a mild collagen deposition, but no important difference between groups was observed in Picrossirius Red staining. n = 4–6 mice/dpi/group. Abbreviations: dpi, days post-infection; GI, gastrointestinal infection; OI, oral infection, N.A., not analyzed. n = 5 mice/dpi/group.
(DOCX)

S4 Table Liver histopathological analysis. Male BALB/c mice were infected with 5x10^4^ tissue culture-derived trypomastigotes forms of *T*. *cruzi* (Tulahuén strain) through gavage (GI) or oral cavity (OI). Livers were harvested at different days post-infection (dpi), fixed and embedded in paraffin. Histological sections were stained by Hematoxylin-Eosin and Picrossirius Red. The table represents degree of inflammation in hepatic tissue. *U*.*i*., uninfected. In hepatic tissue, the inflammatory infiltrates were scored as:-, without infiltrates; +, mild lesions areas; ++, moderate areas of infiltrates; +++, severe areas of infiltrates, ++++ very severe. Thickening of blood vessels walls observed in Hematoxylin-Eosin staining and was corroborated with Picrossirius red technique. n = 3–6 mice/dpi/group. Abbreviations: dpi, days post-infection; GI, gastrointestinal infection; OI, oral infection, N.A., not analyzed. n = 5 mice/dpi/group.
(DOCX)
