# Supplementary figures and images for: Trypanosoma cruzi Infection through the Oral Route Promotes a Severe Infection in Mice: New Disease Form from an Old Infection?
Source: PLoS Negl Trop Dis. 2015 Jun 19;9(6):e0003849. doi: 10.1371/journal.pntd.0003849 (PMC4474863; doi:10.1371/journal.pntd.0003849)

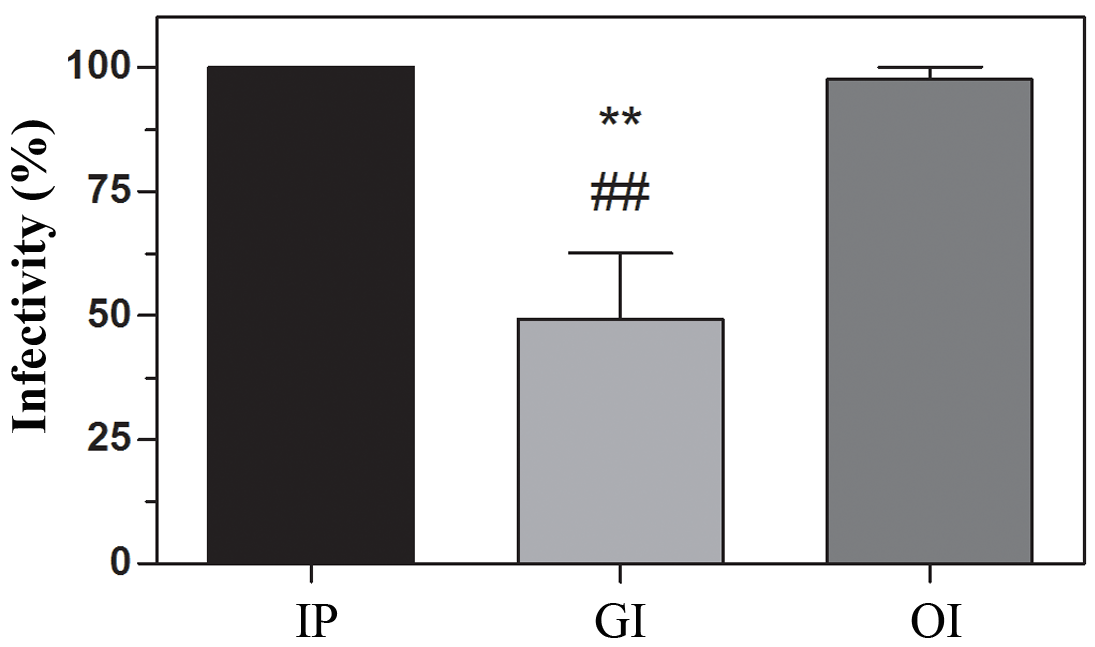

Supplement: S1 Fig — Male BALB/c mice were infected with 5x10^4^ tissue culture-derived trypomastigotes through intraperitoneal (IP), gavage (GI) or oral (OI) inoculation. Infectivity was obtained from the percentage of mice presenting parasitemia over the total number of mice inoculated with parasites. Kruskal-Wallis (Dunn’s post-test) was performed and symbols represent comparison to IP or OI, * and #, respectively. Statistical analysis was performed using GraphPad Prism 5. **/## p = 0.01 (TIF) [file pntd.0003849.s001.tif]

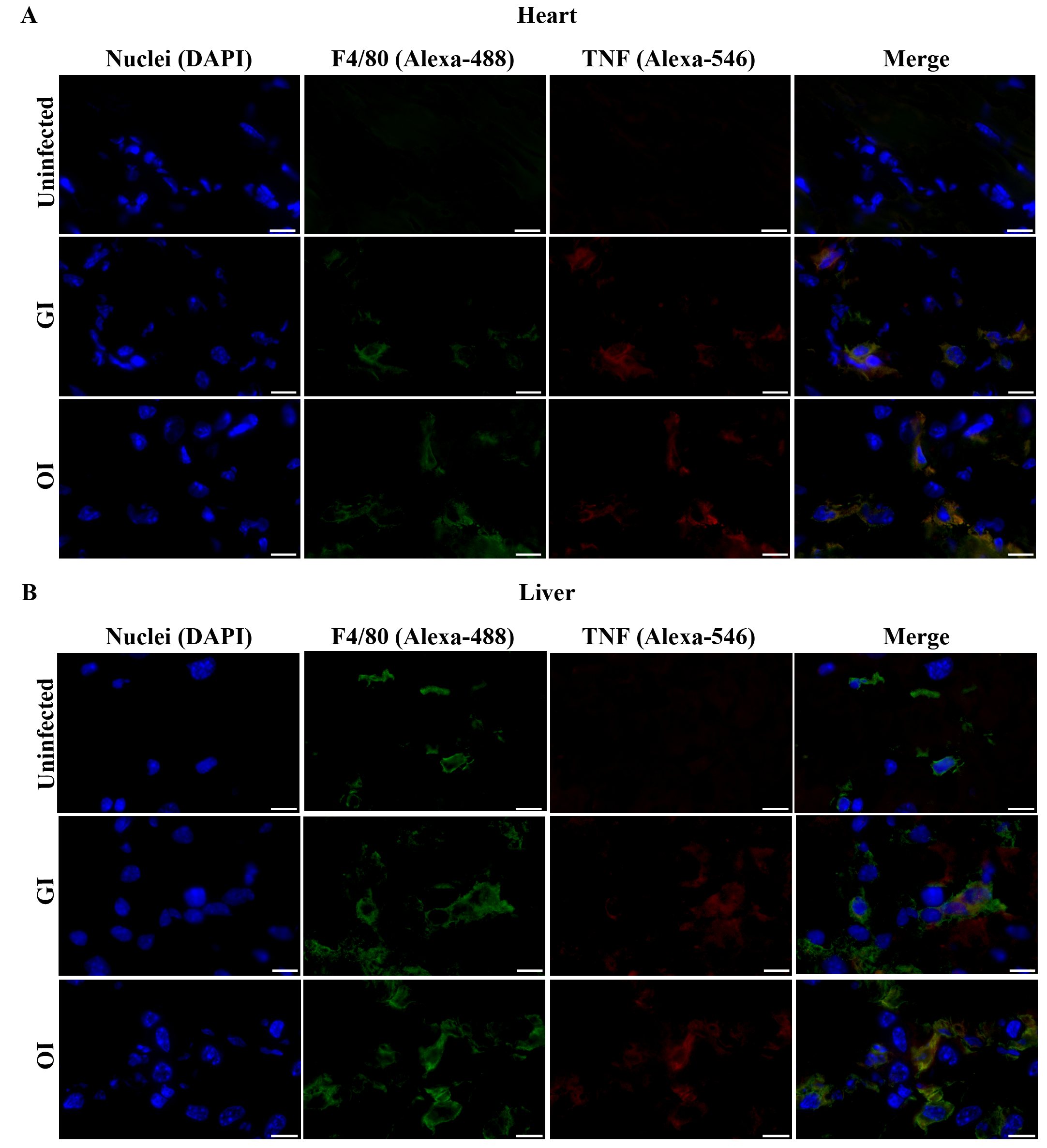

Supplement: S2 Fig — Male BALB/c mice were infected with 5x10^4^ tissue culture-derived trypomastigotes through gavage (GI) or oral (OI) inoculation. A, heart and B, liver tissue cryosections were submitted to double immunostaining for F4/80 (green) and TNF (red). n = 3 mice/group (two sections from each). Bars represent 100 μm. (TIF) [file pntd.0003849.s002.tif]

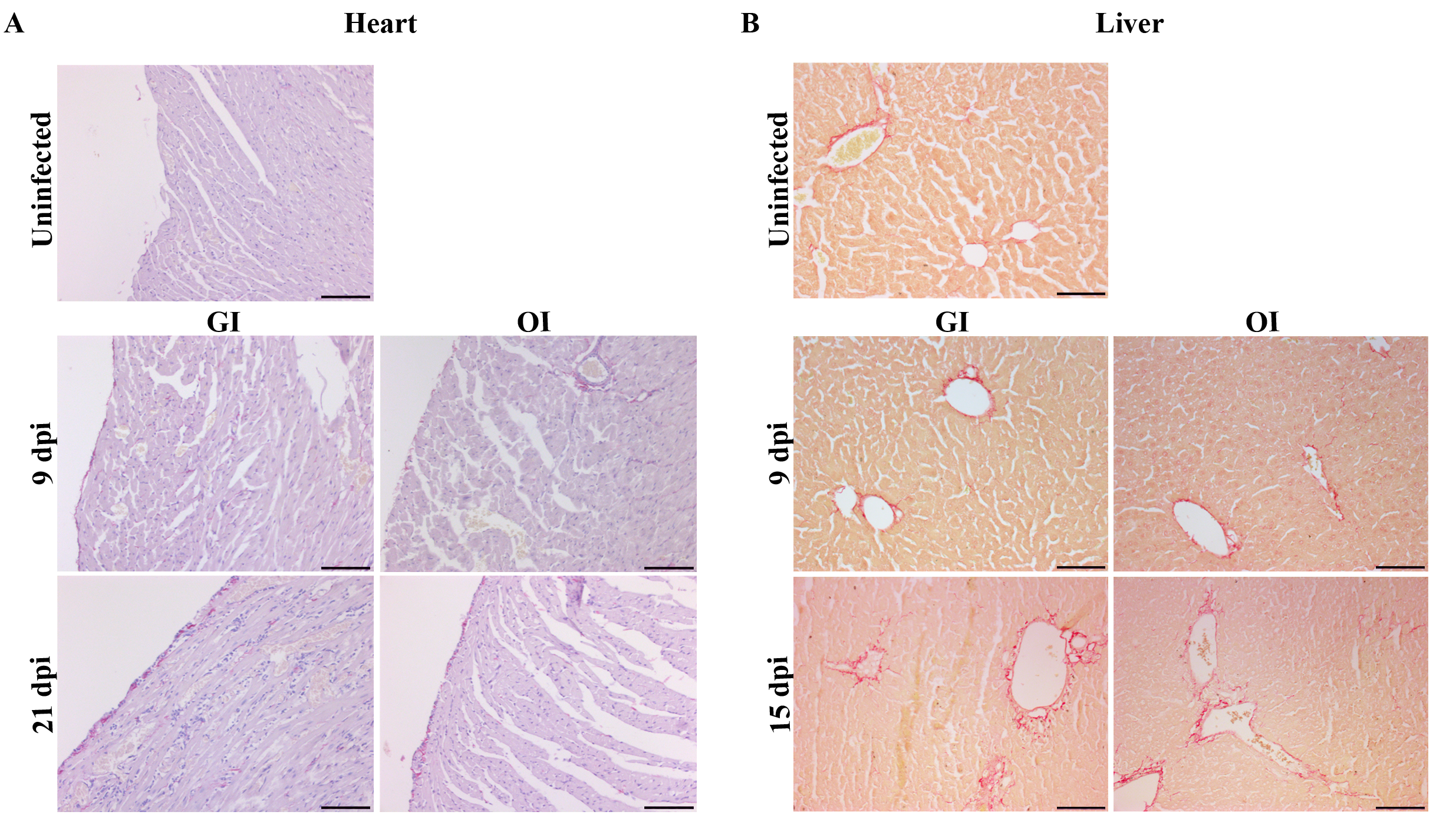

Supplement: S3 Fig — Male BALB/c mice were infected with 5x10^4^ tissue culture-derived trypomastigotes through gavage (GI) or oral (OI) inoculation. Paraffin-embedded sections were stained with Picrossirius to reveal collagen production. A, heart, counterstained with Hematoxylin and B, liver. n = 3–5 mice/group/dpi. Bars represent 50 μm. (TIF) [file pntd.0003849.s003.tif]

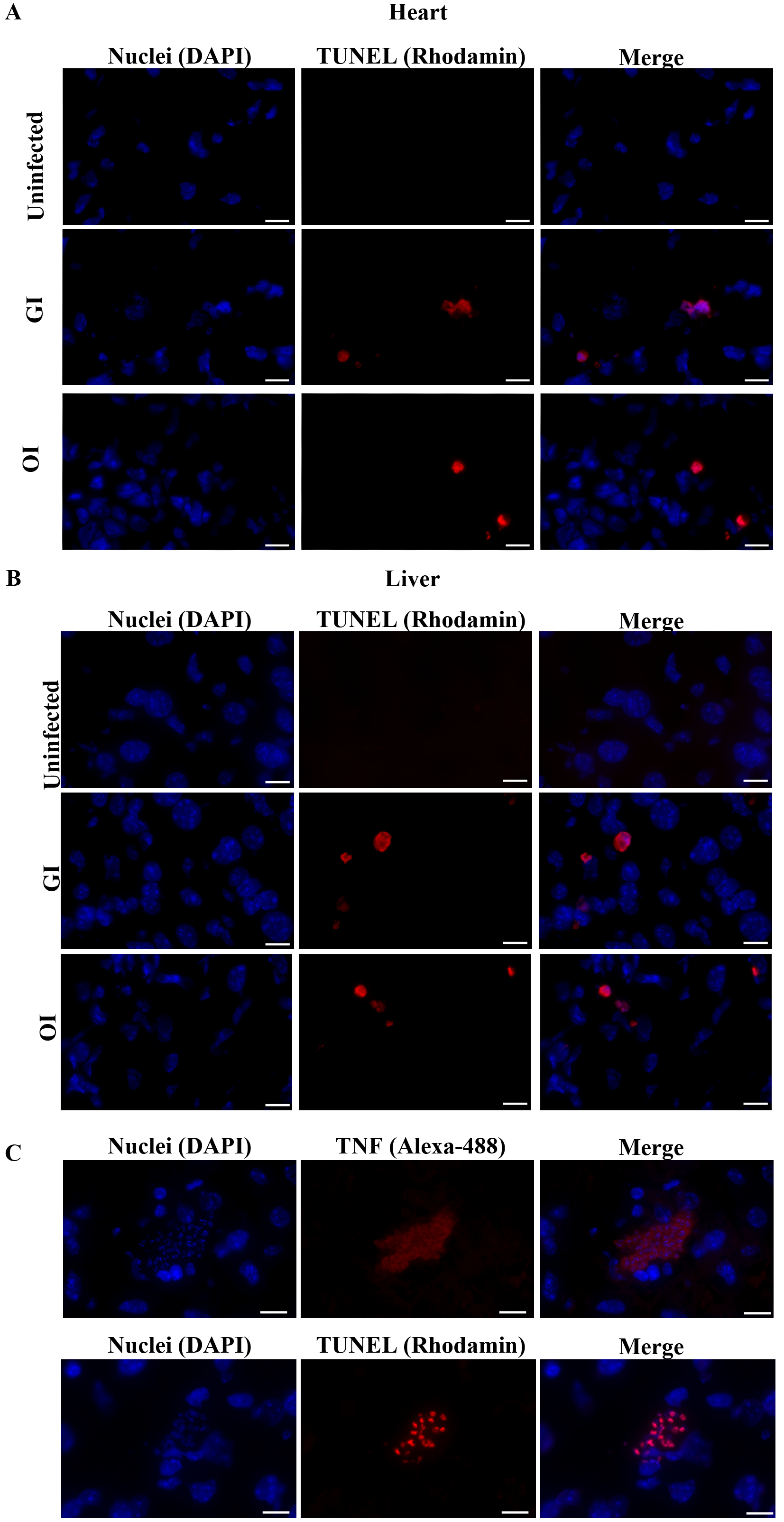

Supplement: S4 Fig — Male BALB/c mice were infected with 5x10^4^ tissue culture-derived trypomastigotes through gavage (GI) or oral (OI) inoculation. TUNEL technique was applied to A, heart and B, liver cryosections for apoptosis detection. C, TNF staining (red) associated with structures that were also related to TUNEL staining (red) within the liver from infected mice. n = 3 mice/group (two sections from each). Bars represent 100 μm. (TIF) [file pntd.0003849.s004.tif]
