# Supplementary material for: Trypanosoma cruzi Infection through the Oral Route Promotes a Severe Infection in Mice: New Disease Form from an Old Infection?
Source: PLoS Negl Trop Dis. 2015 Jun 19;9(6):e0003849. doi: 10.1371/journal.pntd.0003849 (PMC4474863; doi:10.1371/journal.pntd.0003849)
Supplement: S1 Table — Abbreviations: Ig, immunoglobulin; mAb, monoclonal antibody. (DOCX) [file pntd.0003849.s005.docx]

**Table S1. Antibodies applied in immunofluorescence.**

| Molecular specificity | Species specificity | Species origin and immunoglobulin isotype | Stock concentration | Dilution applied | Clone | Manufacturer |
| --- | --- | --- | --- | --- | --- | --- |
| Primary antibodies |  |  |  |  |  |  |
| CD4 | Mouse | Rat mAb IgG2a, κ | 0.5 mg/mL | 1:20 | H129.19 | BD Pharmingen |
| CD8a | Mouse | Rat mAb IgG2a, κ | 0.5 mg/mL | 1:20 | 53-6.7 | BD Pharmingen |
| F4/80 | Mouse | Rat mAb IgG2b | 0.1 mg/mL | 1:50 | CI:A3-1 | Abcam |
| Ly6G | Mouse | Rat mAb IgG2a, κ | 0.5 mg/mL | 1:50 | 1A8 | BD Pharmingen |
| TNF alpha | Mouse/human/Pig/Fish/Cynomologus Monkey/Chicken/Guinea pig/Dog | Rabbit polyclonal IgG | 0.2 mg/mL | 1:25 | Polyclonal | Abcam |
| Secondary antibodies |  |  |  |  |  |  |
| IgG | Rat | Alexa Fluor^®^ 488 goat polyclonal | 2 mg/mL | 1:400 | _______ | Invitrogen |
| IgG | Rabbit | Alexa Fluor^®^ 546 goat polyclonal | 2 mg/mL | 1:400 | _______ | Invitrogen |

Abbreviations: Ig, immunoglobulin; mAb, monoclonal antibody.
