# Supplementary material for: Trypanosoma cruzi Infection through the Oral Route Promotes a Severe Infection in Mice: New Disease Form from an Old Infection?
Source: PLoS Negl Trop Dis. 2015 Jun 19;9(6):e0003849. doi: 10.1371/journal.pntd.0003849 (PMC4474863; doi:10.1371/journal.pntd.0003849)
Supplement: S2 Table — Sequences of each primer, manufacture and used concentration are indicated. Holding stages were at 95°C for 10 minutes for all primers, and cycling stages (40x) varied depending on the target. For IL-10, IFN-γ, TGF-β cycling stage was at 95°C for 15 seconds and at 63°C for 1 minute. For TNF, at 95°C for 15 seconds and 64°C for 30 seconds. HPRT and β-actin cycles were performed in the same conditions of each target. (DOC) [file pntd.0003849.s006.doc]

Table S2. Primers sequences for target mRNAs.

| **Target (mRNA)** | **Forward** | **Reverse** | **Manufacture**  **(Ref Seq Number)** | **Holding and cycling (40x) stages** | **Primer concentration*** |
| --- | --- | --- | --- | --- | --- |
| **IL-10** | ATGGCCTTGTAGACACCTTG | GCTATCGATTTCTCCCCTGTG | IDT (NM_010548) | 95 °C – 10 min  95 °C – 15s  63 °C – 1min | 300nM |
| **IFN-γ** | GCCTAGCTCTGAGACAATGAACGC | CACCATCCTTTTGCCAGTTCCTCCA | IDT (This study) | 95 °C – 10 min  95 °C – 15s  63 °C – 1min | 300nM/300nM |
| **TNF** | GGCAGAAGAGGCACTCCCCCA | TGGTGGTTTGCTACGACGTGGG | IDT (This study) | 95 °C – 10 min  95 °C – 15s  64 °C – 30s | 100nM/100nM |
| **TGF-β** | CCGAATGTCTGACGTATTGAAGA | GCGGACTACTATGCTAAAGAGG | IDT (NM_011577) | 95 °C – 10 min  95 °C – 15s  63 °C – 1min | 300nM |
| **HPRT** | TCCCAGCGTCGTGATTAGCGATG | GGCCACAATGTGATGGCCTCCC | Invitrogen (H6995B01/ H6995B02) | 95 °C – 10 min  95 °C – 15s  63 °C/64 °C – 1min/30s | 300nM |
| **β-actin** | GTCCACACCCGCCACCAGTTCG | ATGCCGGAGCCGTTGTCGAC | Invitrogen (467G05/467G06) | 95 °C – 10 min  95 °C – 15s  63 °C/64 °C– 1min/30s | 300nM |

*20µL final volume of reaction
