# Supplementary material for: Trypanosoma cruzi Infection through the Oral Route Promotes a Severe Infection in Mice: New Disease Form from an Old Infection?
Source: PLoS Negl Trop Dis. 2015 Jun 19;9(6):e0003849. doi: 10.1371/journal.pntd.0003849 (PMC4474863; doi:10.1371/journal.pntd.0003849)
Supplement: S3 Table — Male BALB/c mice were infected with 5x10^4^ tissue culture-derived trypomastigotes forms of T. cruzi (Tulahuén strain) through gavage (GI) or oral cavity (OI). Hearts were harvested at different days post-infection (dpi), fixed and embedded in paraffin. Histological longitudinal sections were stained by Hematoxylin-Eosin and Picrossirius Red. The table represents degree of pericarditis and myocarditis was classified as: +, very mild; ++, mild; +++, moderate; ++++, severe; +++++, very severe. Amastigotes nests were observed inside or next infiltrating areas. Infected mice presented a mild collagen deposition, but no important difference between groups was observed in Picrossirius Red staining. n = 4–6 mice/dpi/group. Abbreviations: dpi, days post-infection; GI, gastrointestinal infection; OI, oral infection, N.A., not analyzed. n = 5 mice/dpi/group. (DOCX) [file pntd.0003849.s007.docx]

**Table S3. Heart histopathological analysis.**

|  | Pericarditis | | Myocarditis | |
| --- | --- | --- | --- | --- |
|  | **GI** | **OI** | **GI** | **OI** |
| 3 | - | - | - | - |
| 9 | + | + | + | + |
| 15 | +++ | +++ | ++ | ++ |
| 21 | +++++ | ++++ | +++++ | ++++ |
| 25 | ++++ | N.A. | ++++ | N.A. |
