# Supplementary material for: Trypanosoma cruzi Infection through the Oral Route Promotes a Severe Infection in Mice: New Disease Form from an Old Infection?
Source: PLoS Negl Trop Dis. 2015 Jun 19;9(6):e0003849. doi: 10.1371/journal.pntd.0003849 (PMC4474863; doi:10.1371/journal.pntd.0003849)
Supplement: S4 Table — Male BALB/c mice were infected with 5x10^4^ tissue culture-derived trypomastigotes forms of T. cruzi (Tulahuén strain) through gavage (GI) or oral cavity (OI). Livers were harvested at different days post-infection (dpi), fixed and embedded in paraffin. Histological sections were stained by Hematoxylin-Eosin and Picrossirius Red. The table represents degree of inflammation in hepatic tissue. U.i., uninfected. In hepatic tissue, the inflammatory infiltrates were scored as:-, without infiltrates; +, mild lesions areas; ++, moderate areas of infiltrates; +++, severe areas of infiltrates, ++++ very severe. Thickening of blood vessels walls observed in Hematoxylin-Eosin staining and was corroborated with Picrossirius red technique. n = 3–6 mice/dpi/group. Abbreviations: dpi, days post-infection; GI, gastrointestinal infection; OI, oral infection, N.A., not analyzed. n = 5 mice/dpi/group. (DOCX) [file pntd.0003849.s008.docx]

**Table S4. Liver histopathological analysis.**

| dpi | Blood peri-vessel Inflammation | | Microgranulomas | | Thrombotic masses | | Collagen production | |
| --- | --- | --- | --- | --- | --- | --- | --- | --- |
|  | **GI** | **OI** | **GI** | **OI** | **GI** | **OI** | **GI** | **OI** |
| 3 | - | - | - | - | - | - | - | - |
| 9 | - | + | - | + | - | - | - | - |
| 15 | ++ | ++++ | ++ | ++++ | ++ | +++ | + | ++ |
| 25 | ++ | N.A. | ++ | N.A. | ++ | N.A. | ++ | N.A. |
